# Cost reduction and quality preservation with digital scanner interfaces for optical coherence tomography

**DOI:** 10.1117/1.JBO.30.11.115005

**Published:** 2025-11-22

**Authors:** Kyoungmo Koo, Lucia Lee, Morgan McCloud, Mark Draelos

**Affiliations:** a University of Michigan, Department of Robotics, Ann Arbor, Michigan, United States; b Duke University, Department of Biomedical Engineering, Durham, North Carolina, United States; c University of Michigan Medical School, Department of Ophthalmology and Visual Sciences, Ann Arbor, Michigan, United States

**Keywords:** optical coherence tomography, digital control, galvanometers, laser, embedded systems

## Abstract

**Significance:**

Optical coherence tomography (OCT) systems are traditionally decomposed into engine and scanner components with an expensive and noise-prone analog interface to communicate the scan pattern between the two. Although simple and convenient, analog signals are susceptible to interference and require expensive hardware to generate with appropriate precision for OCT.

**Aim:**

To overcome these limitations, we implemented a digital interface for our OCT system using low-cost embedded microprocessors and custom PC software, exploiting recent trends toward digital servo drivers for optical scanning.

**Approach:**

Our interface features USB interfacing with a PC for scan pattern download and position feedback upload, 50-kHz communication rate, external triggers with adjustable downsampling, and no external power requirements.

**Results:**

We quantitatively assessed the latency, noise characteristics, and imaging performance of our digital interface in comparison with a conventional analog system that is an order of magnitude more expensive. The signal analysis confirmed that the digital interface reliably transmitted the intended scan pattern to the galvanometer driver and significantly reduced noise in the position feedback signal. High-speed laser trajectory tracking during sparse raster scanning revealed that discrepancies in the analog feedback signal did not reflect actual galvanometer positioning errors; both interfaces achieved equivalent spatial accuracy. Resolution testing demonstrated that both interfaces produced comparable OCT image quality, with no discernible difference up to the system’s resolution limit, whereas reconstruction based on digital interface position feedback outperformed other methods when demanding scan patterns, such as spiral scanning, were applied. To support reproducibility and system integration, we developed a custom printed circuit board (PCB), enabling a compact and robust configuration for future OCT deployments. A simplified version of the firmware is supported by our open-source library *vortex*.

**Conclusions:**

Together, these results demonstrate quantitative and qualitative equivalence of the interfaces, despite an order of magnitude reduction in cost. We released open-source software, PCB schematics, design files, and a bill of materials so that the OCT community can benefit from these improvements and cost savings.

## 1 Introduction

Optical coherence tomography (OCT)^1^^,^^2^ is a non-invasive imaging modality that enables high-resolution cross-sectional visualization of tissue microstructure using low-coherence interferometry.^3^^,^^4^ Since its introduction, OCT has transformed diagnostic and monitoring practices in ophthalmology, where it is now routinely used to evaluate a wide range of ocular diseases,^5^^,^^6^ including age-related macular degeneration,^7^^–^^9^ glaucoma,^10^ and corneal disorders.^11^ Beyond ophthalmology, OCT has also been adopted in dentistry^12^ and dermatology^13^^,^^14^ for its ability to image subsurface structures non-invasively, aiding in the diagnosis of periodontal disease and skin cancer. By providing micrometer-scale resolution^15^^,^^16^ and real-time imaging capabilities,^17^^,^^18^ OCT has become an essential tool in clinical settings,^19^ influencing treatment decisions and contributing to our understanding of disease progression across medical and surgical disciplines.^20^^–^^23^

OCT systems traditionally consist of two system-level components: the engine, which forms the interferometric signal from reference and sample arm light, and the scanner, which controls the spatial distribution of sample arm light. High-quality OCT imaging requires precise synchronization between the engine and scanner so that the captured data are reconstructed at the correct spatial position. The choice of scanner technology and the interface between the engine and scanner is, therefore, a major consideration in OCT system design, and system designers go to great lengths to optimize scanner performance.^24^^–^^27^ A common selection, especially in research and academic settings, is an analog interface in which the engine encodes the target scan position as a voltage that the scanner then senses.^28^^–^^30^ Although simple and convenient, the analog interface is problematic for two reasons. First, analog signals are susceptible to interference and degradation from electromagnetic noise,^31^^,^^32^ temperature fluctuations,^33^ attenuation over long distances, and variations in reference point (i.e., analog zero). The command that the scanner transmits and receives may consequently differ from what the engine expected. Second, analog hardware for this purpose from typical vendors is bulky and expensive yet is still susceptible to the aforementioned issues.

We believe that the OCT community would benefit from an alternative engine–scanner interface that exhibits reduced noise, has 32-bit (16-bit per channel) or better resolution, provides bidirectional communication (position commands and feedback), is created from small-size and low-cost components, and is robust and easy to use. To meet these requirements, we implemented a digital interface for our OCT system using compact and low-cost components from STMicroelectronics’ established STM32 ecosystem of embedded microprocessors, exploiting trends toward digital servo drivers for optical scanning.^34^^,^^35^ Our reference OCT scanner is based on the Mach-DSP servo driver (Pangolin Laser Systems, Sanford, Florida, United States), which supports both analog and digital communication modes. Our interface features USB interfacing with a PC for scan pattern download and position feedback upload, 50-kHz communication rate, external triggers with adjustable downsampling, and no external power requirements. A streaming mode is available for time-unlimited dynamic scan patterns. We include support for this interface in our open-source library *vortex* for creating high-performance OCT engine software in C++ or Python.^36^

We further characterize the latency and noise of this digital interface. High-speed laser trajectory tracking during sparse raster scanning was conducted for direct comparison of actual galvanometer positions. We then evaluate the quality of the resulting OCT images against those obtained from a traditional analog interface that costs 10 times more. This affordable yet advanced digital interface is expected to benefit researchers in biophotonics by enabling the precise signal control and feedback necessary for high-fidelity OCT imaging.

## 2 System Requirements and Device Selection

The digital interface includes a servo driver and galvanometer. *vortex*,^36^ an open-source software utilized for generating images from position and OCT signal data, is employed on the computer for image reconstruction. The microprocessor and digital communication protocols employed to interconnect these devices must be capable of seamlessly executing the following functions:

• Real-time translation of scan trajectories: The microprocessor must convert a predefined scan trajectory—represented as a sequence of spatial positions—into a protocol-compatible bitstream for transmission to the actuator controller.
• Simultaneous bidirectional communication: Accurate feedback from the scanner must be transmitted from the actuator back to the host computer to support the analysis of galvanometer positions. This requires robust conversion of incoming protocol-specific position feedback into a host-readable format.
• Precise synchronization with laser sweep triggers: Each position must be sent from the computer to the servo driver via the microprocessor at a constant interval aligned with the laser sweep trigger pulse.
• Synchronize startup of hardware and software: The system must support synchronized startup and operation among the controller, the scanner hardware, and the host-side image acquisition software. This ensures that position commands and corresponding imaging signals are temporally aligned from the beginning of each scan.
• Stable bidirectional communication rate: The rate of data exchange between the microprocessor and servo driver must not exceed that between the microprocessor and computer. This constraint arises because the former exchange occurs continuously, synchronized with the laser trigger pulse. The computer must always prepare the new scan pattern in advance for transmission from the microprocessor to the servo driver, ensuring the servo driver receives up-to-date position data. Similarly, the microprocessor must complete the transmission of a previous chunk of position feedback to the computer before starting to receive a new chunk of feedback from the servo driver.

**Fig. 1:**
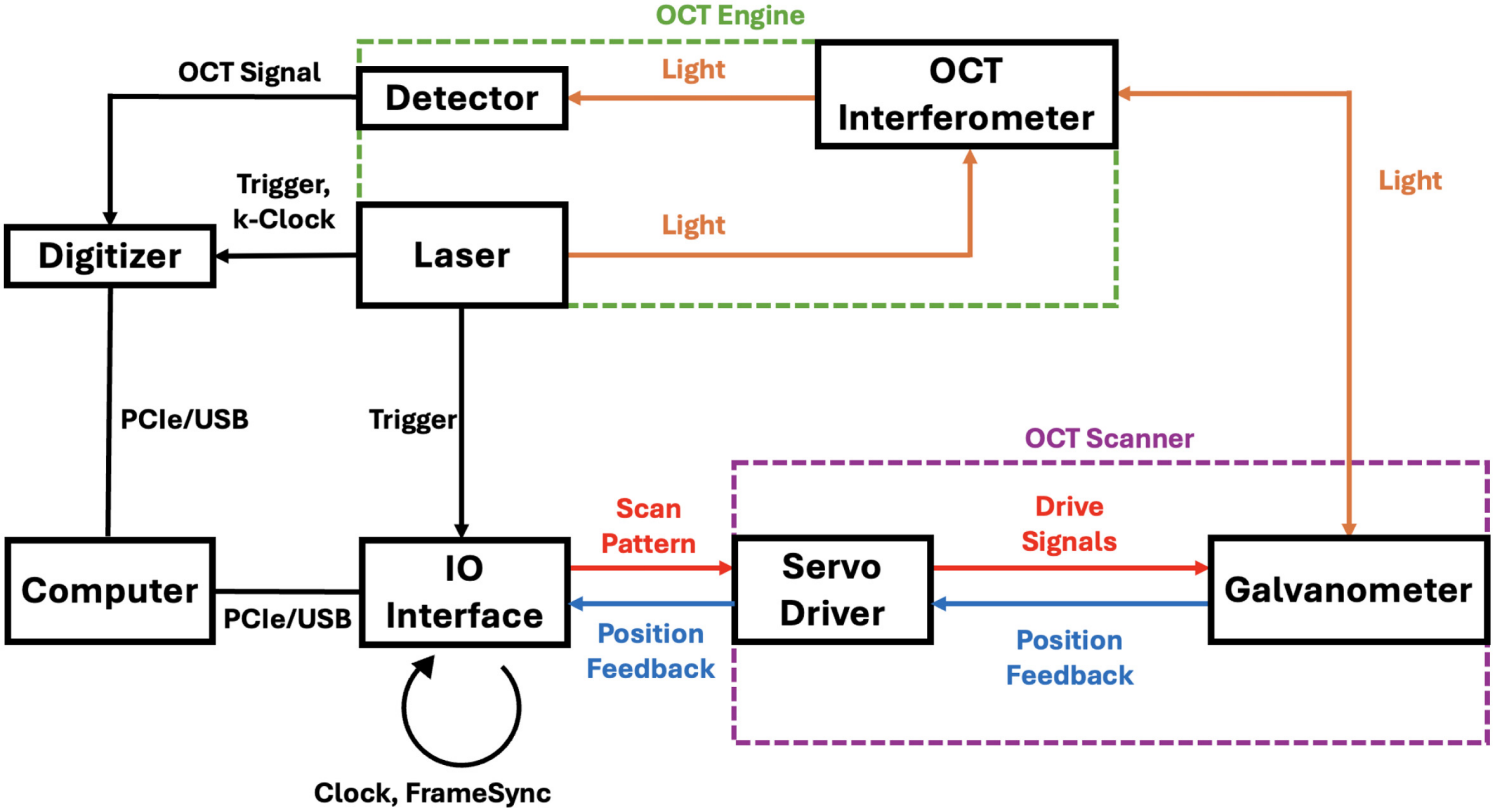
High-level schematic for a prototypical swept-source OCT system. The engine (green dashed box) consists of the laser, detector, and interferometer, which generate and process the optical signals for image formation. The galvanometer scans light across the sample in this reflective sample arm. The laser provides both the trigger and k-clock signals that synchronize the digitizer, which acquires the OCT signal from the interferometer and transfers the digitized data to the computer via PCIe or USB. The IO interface coordinates with the computer to generate the scan pattern and corresponding control signals. The OCT scanner (purple dashed box) comprises the servo driver and galvanometer, which receive drive signals from the IO interface to execute the commanded scan trajectories and return position feedback for analysis.

Based on these criteria, STM32 evaluation boards were chosen for their advanced features, widespread availability, and suitability for real-time embedded applications.^37^ The implementation is compatible across various STM32 platforms, which commonly feature ARM Cortex-M cores, sufficient RAM, and flexible peripheral support for reliable communication and control. The boards support direct memory access (DMA) for multiple peripherals, which alleviates the constraints of a single-core CPU by enabling peripherals to operate independently without CPU intervention. As a result, all peripherals utilized in this study were configured to operate in DMA mode whenever feasible. Furthermore, the boards provide a range of interfaces essential for each specific function above. Communication diagram with our interface integrated is illustrated in Fig. 1.

## 3 System Design

### 3.1 Microprocessor: Servo Driver Link—Serial Audio Interface (SAI)

Common digital standards for controlling galvanometers routinely used in OCT scanning include XY2-100, SL2-100, and the FB4 protocol.^38^^,^^39^ Although these protocols share similar principles, the FB4 protocol was selected for our experiment due to its ability to provide simultaneous high-accuracy position feedback. The FB4 protocol is a public four-wire protocol that includes FrameSync, Clock, TX (transmit), and RX (receive) lines. Each 32-bit data segment on the TX and RX lines comprises 16 bits (2 bytes) of x-position data and 16 bits (2 bytes) of y-position data for the galvanometer. Among the various interfaces supported by the STM32, the serial audio interface was found to be compatible with the FB4 protocol when the communication lines were properly configured. Table 2 in Appendix A (Sec. 8.1) provides the specific functions and settings used to configure STM32 SAI for FB4 compatibility.

### 3.2 Computer: Microprocessor Link—Universal Asynchronous Receiver/Transmitter (UART) and Serial Peripheral Interface (SPI)

Continuous operation of SAI transmission (TX) and reception (RX) necessitates careful timing coordination between the computer and the STM32 board to prevent data interruption or loss. TX and RX buffers within the SAI are divided into two halves, synchronized such that as TX completes transmitting data in one half, RX concurrently finishes receiving data in the corresponding half. To avoid conflicts, the computer must operate on the buffer half not currently accessed by SAI. Two communication architectures were developed for interfacing the STM32 with the computer system. The first, used in this study, combines UART-based signaling with SPI-based data exchange, supporting high-throughput acquisition. The second employs UART alone for both control and data transfer, offering a simplified alternative. The UART-only configuration is implemented in the digital mode of the open-source *vortex* software. Table 3 of Appendix B (Sec. 8.2) provides detailed descriptions of the functions and libraries used on the computer and STM32 to implement this link.

### 3.3 Synchronization of Laser Trigger and SAI

It is important to make sure that each time the laser trigger signals a pulse, a 32-bit position value is delivered to the servo driver. Our *vortex* software, employed for image reconstruction, requires that the photoreceiver’s data are synchronized with the scan position data it sends out to the galvanometer up to a fixed known offset. This synchronization allows for the precise pairing of these data points to reconstruct each A-scan accurately. When the laser trigger frequency exceeds the SAI channel’s frame rate, the scan pattern must be downsampled accordingly. The timer (TIM) peripheral on the STM32 board is used to synchronize the SAI channel with the laser trigger signal with configurable downsampling. Appendix C (Sec. 8.3) provides detailed equations for determining the prescaler and counter period of the STM32 TIM interface to achieve a target downsampling factor.

### 3.4 Achievable Speed of Communication with Margin for Robustness

**Fig. 2:**
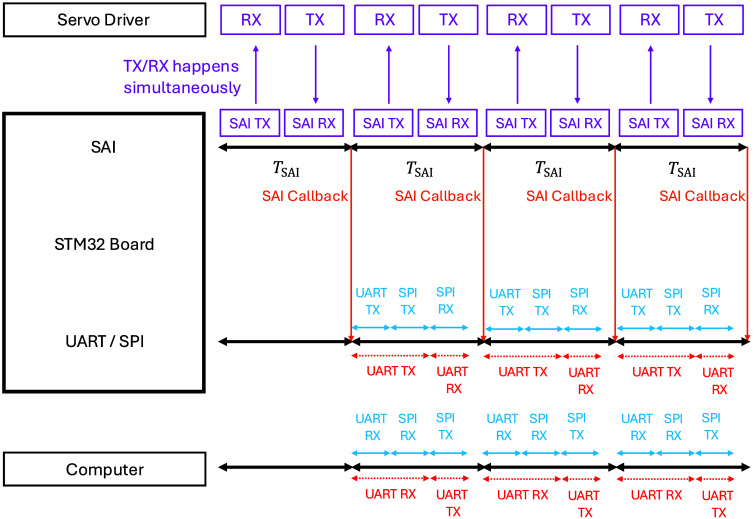
Timing diagram of the system. Continuous exchange of scan patterns and position feedback occurs between the STM32 and the servo driver via the SAI/FB4 protocol. The diagram includes the notification process via UART that the chunk of SAI transmission is complete, and data transmission/reception via UART+SPI (blue) or UART-only (red) configuration.

Continuous bidirectional communication between the STM32 microprocessor and the servo driver is facilitated via the SAI/FB4 protocol, enabling synchronized transmission of scan patterns and reception of position feedback. Upon completion of transmission and reception in each buffer half, a callback is triggered, prompting the STM32 to notify the computer via UART. Subsequently, the computer, acting as the master, begins sending the clock signal, and the STM32 board sends the recently received position feedback from the servo driver back to the computer. Figure 2 presents the system-level timing diagram corresponding to this process.

In the UART+SPI configuration, used in this study, the computer subsequently initiates SPI communication as the bus master. The computer begins sending the clock signal, and the STM32 board sends the recently received position feedback from the servo driver back to the computer. After receiving the position feedback, the computer transmits data via the SPI bus into half of the scan pattern buffer in the STM32 board that was just sent to the servo driver. In the UART-only configuration, both notification and data exchange occur over UART. Position feedback is received continuously after notification by the computer, which then updates the scan pattern buffer via UART in the same half-cycle structure.

A crucial assumption of this timing diagram is that the time spent on TX/RX for half of the buffer in the SAI part must be longer than the preparation time. The preparation time includes notification via UART and the transmission and reception of each chunk via the UART or SPI bus. Equation 1 is derived considering this timing requirement. $T_{\mathrm{SAI}}$ represents the time used for TX/RX of each chunk of the buffer. $L_{\mathrm{buffer}}$ denotes the length of the buffer, defined as the number of positions in the buffer. $T_{\mathrm{UART}}$ indicates the time used for notification to the computer via UART. $f_{\mathrm{SAI}}$ represents the frame frequency of SAI, and $f_{\mathrm{UART}/\mathrm{SPI}}$ denotes the baud/bit rate of the UART/SPI bus

$$T_{\mathrm{SAI}}=\frac{0.5\cdot L_{\mathrm{buffer}}}{f_{\mathrm{SAI}}}>T_{\mathrm{UART}}+\frac{0.5*32*\cdot L_{\mathrm{buffer}}}{f_{\mathrm{UART}/\mathrm{SPI}}}\cdot 2.$$ (1)

Rearranging this equation, we arrive at the following constraint:

$$T_{\mathrm{UART}}<L_{\mathrm{buffer}}\cdot (\frac{0.5}{f_{\mathrm{SAI}}}-\frac{32}{f_{\mathrm{UART}/\mathrm{SPI}}}).$$ (2)

It is evident that, assuming a constant delay for the UART interface, the SAI frame frequency can be augmented as the maximum achievable baud/bit rate of UART/SPI increases with the enlargement of the buffer length. However, both parameters are constrained by the limited capacity of the STM32 board’s SRAM and the restricted speed of the computer’s UART or SPI interface, such as a USB-UART or USB-SPI adapter.

For our specific UART+SPI configuration, which was used for data collection in this study, UART notification incurs an initial delay of 15 ms, regardless of the buffer length being transmitted. The frame frequency of SAI is 50 kHz, downsampled by a factor of 4 from the laser trigger at 200 kHz, and the SPI baud rate is set to 15 Mbps. The buffer should contain at least 2000 positions. For robustness with a margin of ∼50%, it is recommended that the buffer contain more than 4000 positions.

For the UART-only configuration, when using the STM32 Nucleo-U545RE-Q evaluation board, equipped with the ST-Link V3 interface—designed for higher-speed UART transmission—the system can operate up to 192 kHz, which is limited by the SAI peripheral of the board. The UART link itself supports read and write operations up to 220 kHz when operated independently. However, due to the SAI bottleneck, the trigger signal must still be downsampled. Higher laser frequencies could be accommodated by adjusting the downsampling ratio.

### 3.5 PID Parameters Tuning

Proportional, integral, derivative (PID) control parameters were utilized for the control of the galvanometers in the MachDSP servo driver. Though the manufacturers distribute the galvanometers already tuned, we used their complementary software application to further configure our system. The software provides error proportional gain and the error integral gain, but not an error derivative gain. Instead, there is position derivative gain tuning, which is obtained by differentiating the position signal (low-frequency damping gain) or integrating the drive current (high-frequency damping gain). We only needed to adjust the error proportional gain and position derivative gain from the original parameters to increase precision and efficacy. This manual tuning procedure is outlined in Sec. 9 of the ScannerMax User Manual.^40^

## 4 Methods

To demonstrate this approach to digital OCT scanner control, we selected the STM32 Nucleo-L476RG evaluation board due to its advanced features and applicability. It is equipped with an ARM Cortex-M4 32-bit RISC core capable of running at speeds up to 80 MHz and includes 128 kB of RAM. We evaluated our digital interface for OCT imaging with regard to its reduction of noise in the position feedback signals and its effect on image quality as compared with a PCIe-6363 analog interface (National Instruments, Austin, Texas, United States). The Nucleo-L476RG and PCI-6363 retail for ∼$15 and $2500 USD, respectively, at the time of writing. We selected a downsampling factor of 4 for the digital interface to satisfy the throughput requirements of Eq. (2).

### 4.1 OCT System

**Fig. 3:**
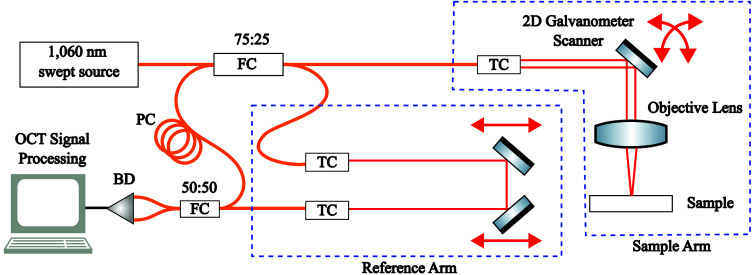
Schematic of the optical coherence tomography (OCT) system. The laser output is split by a fiber-based 75:25 coupler to direct light toward the sample and reference arms. The sample arm includes triple collimators (TC), a 2D galvanometer scanner, and an objective lens to steer and focus the beam onto the sample. The reference arm comprises a pair of triple collimators (TC) and incorporates both fiber and free-space components, with retroreflective mirrors mounted on a motorized translation stage for precise path length adjustment. Light returning from both arms is recombined at a 50:50 coupler and directed to a balanced detector (BD) to suppress common-mode noise and enhance signal quality. The detected signals are then processed by the OCT software on a computer workstation. PC: polarization controller, FC: fiber coupler.

Figure 3 illustrates the optical layout of our OCT system. We used our lab standard OCT system built with a 200-kHz 1060-nm swept source laser (Thorlabs, Newton, New Jersey, United States) which illuminated a Mach–Zehnder interferometer. With 100-nm optical bandwidth, this system has a theoretical axial resolution of 5  μm in air. A 1-GHz balanced detector (Thorlabs) was used to measure the interferometric signal, which was subsequently detected using a $1.0\;\mathrm{GS}\;s^{-1}$ digitizer (ATS-9364, AlazarTech, Pointe-Claire, Quebec, Canada). We acquired images using a telecentric scanner with a 47.7-mm working distance, Airy radius of 27  μm over a 32-mm field of view. Beam scanning was performed using a Saturn 1B dual-axis galvanometer scanner (ScannerMax, Orlando, Florida, United States), controlled via a servo driver. To minimize electromagnetic interference in the analog interface, a 12-in. RG58 BNC coaxial cable was used to connect the servo driver and PCIe-6363 through a BNC-2110 (National Instruments; Austin, Texas, United States) breakout adapter. The BNC-2110 analog terminals were configured in “floating” mode. We used linear and switching power sources for both analog and digital interfaces. Both power sources supply a constant ±24  V to the servo driver, but the switching power supply introduces greater noise and voltage ripple compared with the linear power supply. The MachDSP servo driver uses PID control for galvanometer positioning, with tunable parameters such as error proportional gain and position derivative gain. Precise control was achieved by manually adjusting only the error proportional gain, low-frequency damping gain, and high-frequency damping gain, as guided by the ScannerMax User Manual (Sec. 3.5).^40^ OCT signal generation and processing were performed with custom GPU-accelerated software written in Python using *vortex*.^36^

### 4.2 Characterization of Galvanometer Delay

Inevitable discrepancies arise between the commanded and sensed positions of the galvanometer, including interface errors (e.g., noise), fixed response delays, and galvanometer tracking errors.^41^ The magnitude of these errors not only introduces performance degradation into the generated image but also serves as a metric for evaluating the superiority of different systems. We, therefore, performed imaging with the same scan waveform (512  px×512  px *en face* resolution with 1.000 deg amplitude) separately using the analog and digital interfaces. To isolate the effect of interface errors, we corrected and fixed response delays and used identical galvanometer tunings. We determined the response delay by comparing the actual and target positions and selecting the delay that produced the minimum root mean square error (RMSE).

### 4.3 Characterization of Signal Noise

The performance of the galvanometer is evaluated via three methods: Scan pattern applied via a digital interface and position feedback measured via a digital interface [digital write digital read (DWDR)], applied via a digital interface and measured via an analog interface [digital write analog read (DWAR)], applied via an analog interface and measured via an analog interface [analog write analog read (AWAR)]. The analog write, digital read configuration was not implementable because the servo driver’s digital port did not output position feedback when accepting analog input commands. After excluding the effects of time delay, errors attributed solely to the interface were quantified by calculating the mean absolute error (MAE), RMSE, and standard deviation (SD) between the target and actual galvanometer positions. Both analog and digital control modes were tested under identical conditions using a 512×512  px *en face* raster scan with a 1.000 deg amplitude.

### 4.4 Characterization of Actual Galvanometer Position

**Fig. 4:**
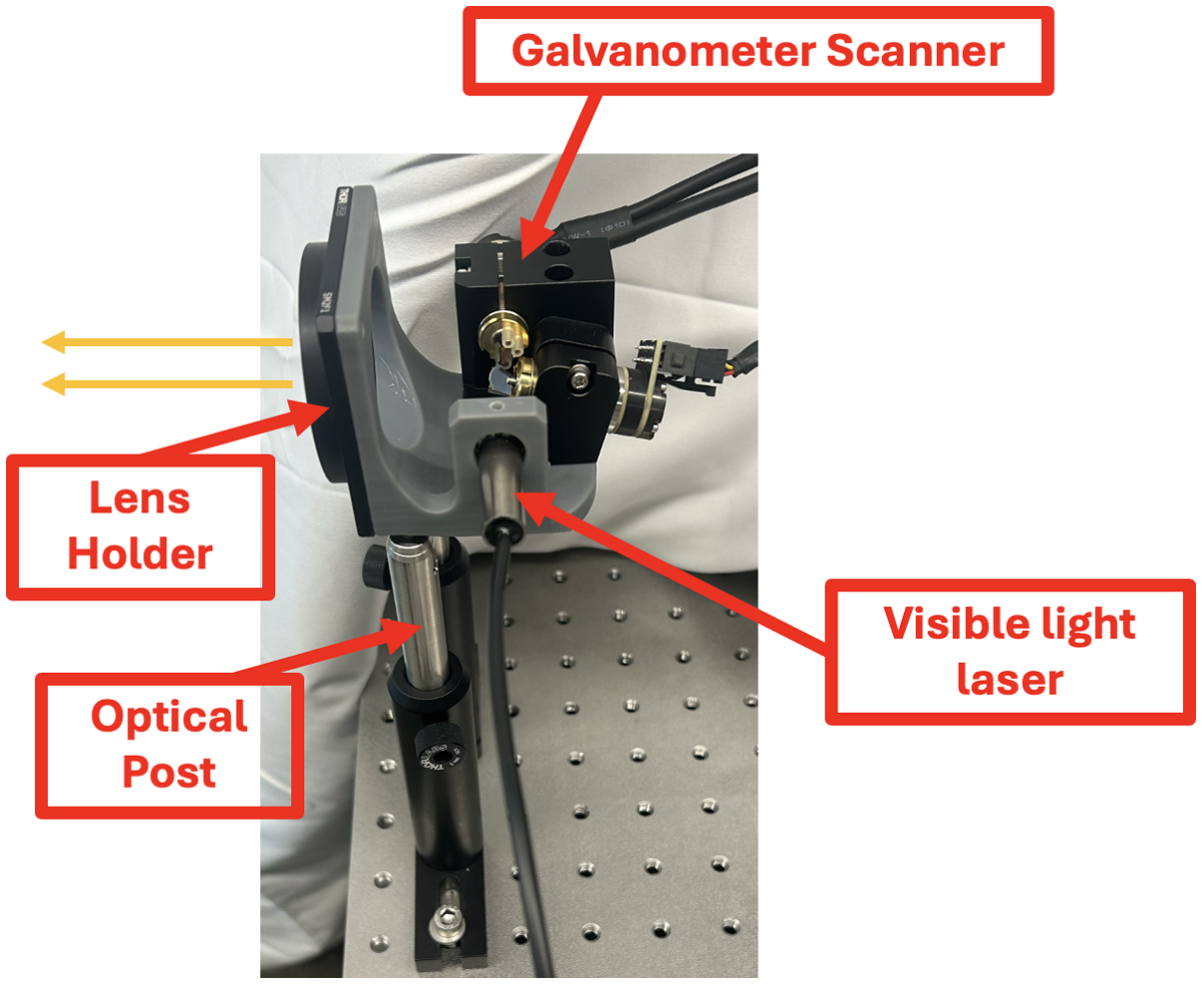
Experimental setup highlighting the main optical components: a galvanometer scanner for beam steering, a lens mount without a lens to preserve beam collimation, an optical post for mechanical support, and a visible light laser for light delivery. The orange arrows indicate the direction of light propagation.

To directly assess whether position feedback noise manifests in the laser trajectory, we performed an additional validation experiment using a sparse raster scan. Both analog and digital control interfaces were tested under identical conditions with a 512×16-px *en face* resolution and a 1.000-deg amplitude scan projected onto a paper screen. A 650-nm, 1-mW visible light alignment laser (VLM-650-03-LPT, Quarton Inc.) was used in place of the OCT laser to facilitate direct observation of the beam path, and the objective lens was removed. The target was positioned at a distance of 1.72 m, sufficient to spatially separate adjacent scan lines and enable clear visualization of the laser trajectory. A Blackfly S BFS-U3-23S3C camera equipped with a 12 mm/f1.8 Edmund Optics C-mount lens (model 58-001) was used to image the scan pattern. The camera was configured to operate at 1 frame per second (FPS) with an exposure time of 10 ms to capture sharp, high-resolution images of the accumulated laser trajectory segments for comparison of scan fidelity between the two control interfaces. Figure 4 illustrates the detailed experimental setup used in the study.

The actual beam width observed in each segment can be considered as the combination of two components: the positional uncertainty of the galvanometer system and the intrinsic beam spread determined by the laser’s spot size. As the optics are identical in both analog and digital configurations, the spot radius remains constant across interfaces. Therefore, the difference in measured beam width between the two systems predominantly reflects differences in positional accuracy, with the analog interface expected to have a longer beam width considering the higher feedback noise. For comparison of the beam width of the two interfaces, 30 images were acquired from each interface. From these, eight image pairs containing corresponding scan segments from both analog and digital modes were selected for analysis. For each segment, beam thickness was quantified by sampling optical intensity along 100 lines perpendicular to the segment. Full width at half maximum (FWHM) was calculated by isolating a 100-px window centered around the peak intensity and identifying the region where the signal remained above a threshold, defined as the midpoint between the maximum and minimum intensity within the window.

### 4.5 Characterization of Image Quality

The USAF-1951 resolution target was used to compare the resolution limits of images acquired through the analog and digital control interfaces. The target was positioned 47.7 mm from the laser source at the scanner’s focal plane, with its surface oriented perpendicular to the laser beam and carefully aligned to center the pattern within the field of view. To prevent sensor saturation and ensure consistent exposure, a neutral density (ND) filter was placed in the optical path during image acquisition.

For quantitative assessment, we acquired OCT data over an ∼0.8×0.8  mm field of view at 512×512  px to capture groups 4 and 5 of the USAF 1951 resolution target. This sampling corresponds to a lateral step size of ∼1.56  μm, yielding more than 18 px across the Airy disk radius (27  μm) of our system. This level of oversampling ensures sufficient spatial resolution to resolve fine structures and comfortably satisfies the Nyquist criterion. We obtained five volumes each with analog and digital interfaces without moving the target. We averaged these volumes together, generated a maximum intensity projection, and extracted profiles across the elements.

For qualitative assessment, we also acquired 3D OCT images over a wider 3.2×3.2  mm field of view using the same pixel density (512×512  px). This configuration, applied to both a multilayered pile of leaves (organic sample) and a printed circuit board (PCB) (artificial sample), corresponds to a lateral step size of ∼6.25  μm. This setup remains oversampled, providing roughly 4.5 px across the system’s Airy disk diameter and preserving fine structural details for qualitative comparison.

To evaluate the impact of galvanometer position feedback accuracy on OCT image reconstruction, a constant linear velocity spiral scan (CLV-SC) experiment was conducted using a USAF 1951 resolution target. The spiral scan was chosen to represent a trajectory that is both efficient and dynamically demanding. All experimental conditions—including the optical setup, acquisition parameters, synchronization scheme, and hardware configuration—were identical to those used in the first experiment, with the only difference being the scan trajectory itself.

## 5 Results

### 5.1 Measured Galvanometer Delay

The galvanometer delay is expected to be consistent across both interfaces, as it is primarily governed by the physical response time of the galvanometer rather than the interface used to deliver the scan pattern. For the analog interface operating at 200 kHz, the optimal delay was 28 samples (140  μs). For the digital interface operating at 50 kHz, the optimal delay was 8 samples (160  μs). This result is consistent with expectations, as the observed delay difference of 20  μs corresponds exactly to the time resolution of the digital interface. The results are illustrated in Appendix D (Sec. 8.4).

### 5.2 Galvanometer Position Feedback Analysis

**Fig. 5:**
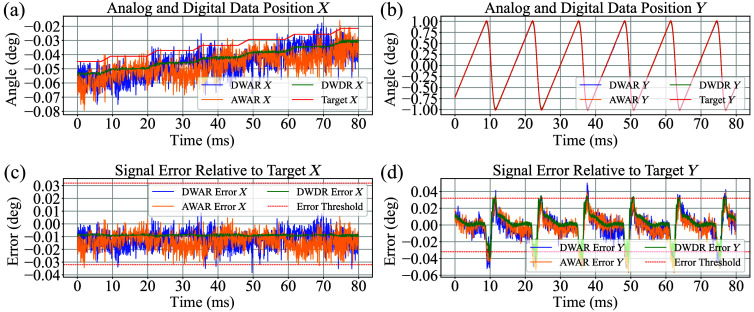
Galvanometer response with linear power source. (a) Position *X* comparison of analog and digital signal paths against the target trajectory. (b) Position *Y* comparison of analog and digital signal paths against the target trajectory. (c) Error relative to target *X*, with ±0.032-deg threshold indicated. (d) Error relative to target *Y*, also including ±0.032-deg error thresholds.

**Fig. 6:**
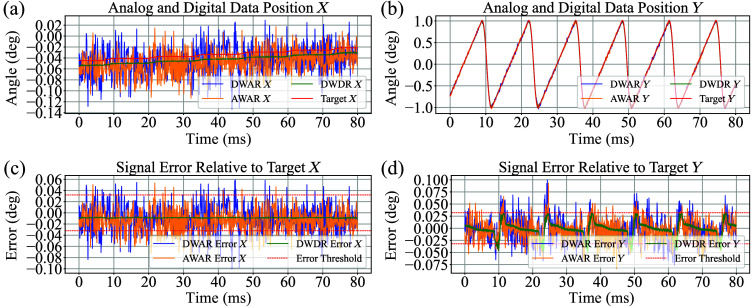
Galvanometer response with switching power source. (a) Position X comparison of analog and digital signal paths against the target trajectory. (b) Position Y comparison of analog and digital signal paths against the target trajectory. (c) Error relative to target X, with ±0.032-deg threshold indicated. (d) Error relative to target *Y*, also including ±0.032-deg error thresholds.

For errors attributed solely to the interface, after excluding those caused by time delay, MAE, RMSE, and SD were calculated and are shown in Table 1. A total of 83,840 pairs (512×512-px resolution waypoints, downsampled by 4) of target and actual galvanometer position data were used for comparison, which constitute one full raster scan. Both active and inactive segments of the galvanometer scanning pattern were included in the error analysis. The active scanning phase spanned 10.24 ms (512 px), whereas the turnaround (inactive) segment among successive scans lasted 2.86 ms (143 px) before downsampling. Digital write/digital read (DWDR) consistently outperformed all other configurations across all metrics, irrespective of axis (X or Y) and power source type (linear or switching). In contrast, digital write/analog read (DWAR) and analog write/analog read (AWAR) exhibited nearly identical performance under both power conditions, generally resulting in approximately twofold higher errors compared with DWDR. Reading through the analog interface resulted in greater errors when a switching power source was applied, indicating increased sensitivity to power conditions. In contrast, the digital interface demonstrated more stable performance, with minimal dependence on the type of power source. These trends are consistent with the results presented in Figs. 5 and 6, which corroborate the quantitative findings summarized in the table. The error threshold of 0.032 deg was derived by calculating the arctangent of the galvanometer’s minimum resolvable displacement, 27  μm for our setup, divided by the distance between the galvanometer and the specimen, 47.7 mm for our setup. This value represents the smallest angular deviation that would produce a physically discernible shift at the target plane and thus reflects the effective resolution limit of the system. Any error below this threshold is considered indistinguishable from intrinsic system resolution and unlikely to affect the practical performance of our system.

**Table 1:** Quantitative comparison of galvanometer tracking performance across read/write configurations using linear and switching power sources.

Mode	Power	X-MAE	X-RMSE	X-SD	Y-MAE	Y-RMSE	Y-SD
DWDR	Linear	**8.8**	**9.7**	**4.4**	**8.9**	**15.9**	**15.9**
DWAR	Linear	16.1	19.3	15.0	12.0	16.4	16.4
AWAR	Linear	17.1	20.5	16.2	11.3	16.3	16.1
DWDR	Switching	**8.3**	**8.8**	**3.0**	**9.2**	**13.9**	**13.8**
DWAR	Switching	23.1	28.8	26.3	20.4	26.0	26.0
AWAR	Switching	22.7	28.3	25.6	19.1	24.3	24.0

DWDR, digital write/digital read; DWAR, digital write/analog read; AWAR, analog write/analog read; MAE, mean absolute error; RMSE, root mean square error; SD, standard deviation. Values in millidegrees (m°). Bold indicates the lowest error per column.

### 5.3 Experimental Validation of Galvanometer Position

**Fig. 7:**
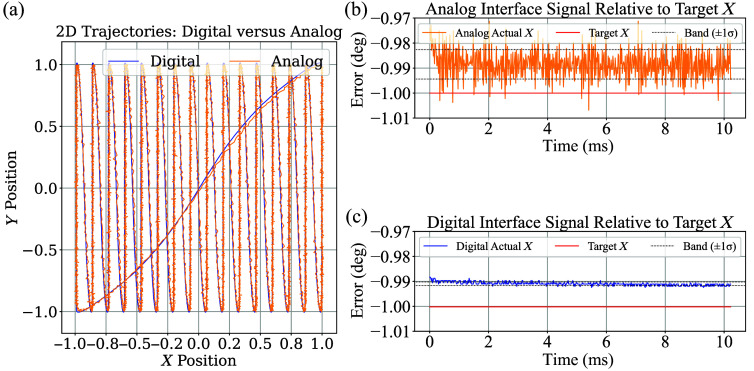
(a) Two-dimensional trajectory obtained from the analog (orange) and digital (blue) interface position feedback signals under a sparse raster scan. (b) Analog position feedback (orange) and corresponding target signal (red), with dotted lines indicating ±1 standard deviation. (c) Digital position feedback (blue) with the same target reference and band definition. The estimated width of the band for each segment is defined as twice the standard deviation of the signal over time.

To measure the error present in the laser beam path, the switching power source was used, and 10,480 pairs (512×16  px resolution waypoints) of galvanometer positions were analyzed for both interfaces. Both active and inactive segments of the galvanometer scanning pattern were included in the error analysis. The active scanning phase spanned 10.24 ms (512 px), whereas the turnaround (inactive) segment among successive scans lasted 2.86 ms (143 px). Figure 7 illustrates the standard deviation of position feedback signals for both the analog and digital interfaces. The standard deviation was 0.0055 deg for the analog system and 0.0005 deg for the digital system, indicating a markedly reduced positional variation in the digital feedback, according to position feedback signals.

**Fig. 8:**
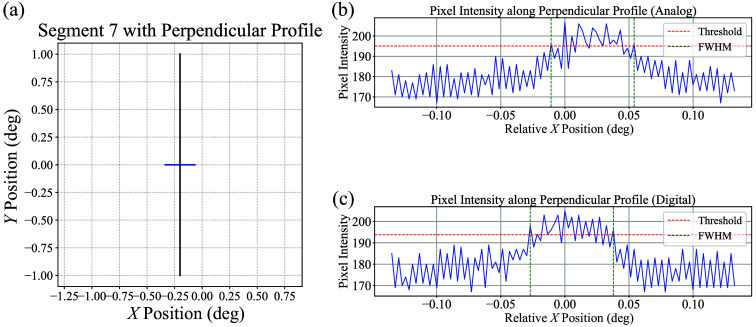
Visualization of FWHM analysis from segment 7. (a) Raster trajectory of segment 7 is shown in the X−Y scan space, with the blue line indicating the perpendicular profile selected for analysis. (b) and (c) Pixel intensity profiles along the perpendicular line for the analog (b) and digital (c) interfaces, respectively. The FWHM was computed using a threshold set to the midpoint between the maximum and minimum intensity values. Green lines mark the start and end positions of the FWHM band.

**Fig. 9:**
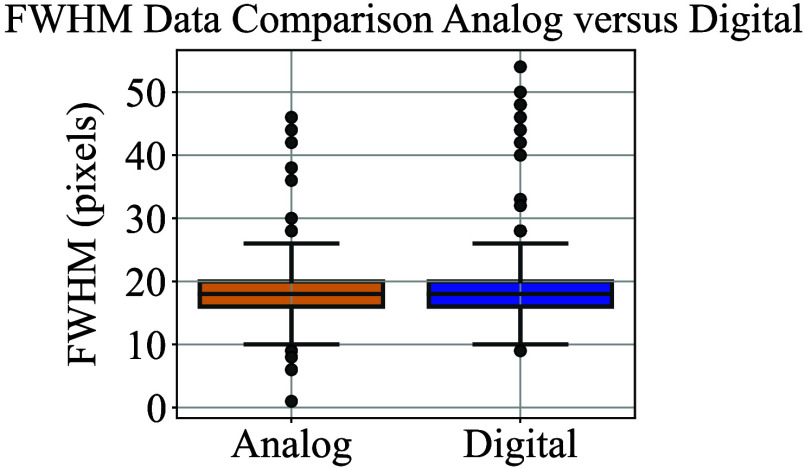
Box plots of FWHM values computed from 800 perpendicular line samples across matched scan segments acquired with analog and digital galvanometer control. Each box represents the interquartile range (IQR), with the median marked within the box and whiskers extending to 1.5× IQR. Outliers are shown as individual points. The analog and digital interfaces showed comparable distributions, with nearly identical medians (18.0 px) and interquartile ranges (16.0 to 20.0 px).

To determine the physical size represented by each camera pixel, a calibration image was taken using a circular trajectory with a known angular radius of 0.1000 deg. Based on four calibration images acquired under both analog and digital control modes, the camera pixel resolution was measured to be 0.0030 deg. The FWHM of each segment was computed from each line profile. As an illustrative example, the analysis of a single profile extracted around segment 7 is presented in Fig. 8. As shown in Fig. 9, both control modes exhibited comparable FWHM distributions. The analog interface yielded a mean FWHM of 18.9 px (standard deviation: 3.7 px), whereas the digital interface produced a mean of 19.1 px (standard deviation: 4.4 px). The interquartile range (IQR) was identical for both groups (Q1: 16.0 px, median: 18.0 px, Q3: 20.0 px), indicating a similar central tendency and dispersion.

To determine whether any statistically significant difference existed between the two groups, a two-sided Mann–Whitney *U* test was performed. The resulting *p*-value (p=0.66) indicated no significant difference in FWHM distributions between analog and digital control modes. These findings suggest that the galvanometer position noise levels are statistically indistinguishable between the two interfaces, supporting their equivalence in positional precision under experimental conditions.

### 5.4 Quantitative Imaging Results

**Fig. 10:**
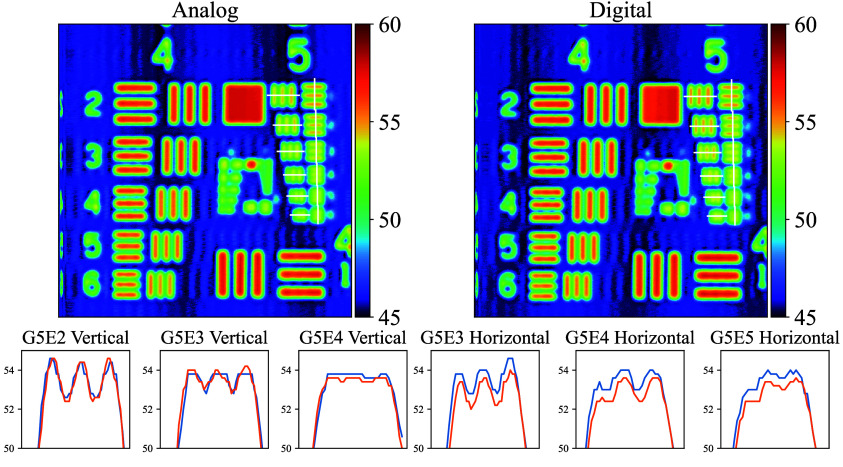
Comparison of analog (orange) and digital (blue) interfaces scanning groups 4 and 5 of a USAF target and plots through specific elements. Both interfaces resolve down to group 5 element 3 or 4 for horizontal and vertical line pairs, respectively, demonstrating equivalent performance to the resolution of our scanner. Note the scanner flyback artifact on the left edge because we disabled *vortex*’s galvanometer latency correction for these experiments.

Both interfaces successfully resolved all elements of group 4 of the USAF 1951 target, for both horizontal and vertical line pairs, as can be seen in Fig. 10. Consequently, our focus shifted to a detailed analysis of the elements in group 5. The elements that showed the resolution limits for analog or digital interface were elements 2 to 4 for horizontal line pairs and elements 3 to 5 for vertical line pairs. For horizontal line pairs, element 2 exhibited clear contrast for both the analog and digital interfaces. However, this contrast diminished progressively for element 3 and ultimately failed to produce meaningful contrast for element 4. A similar phenomenon was observed on the vertical side, starting with a resolvable element 3 and progressing to unresolvable element 5. Overall, line plots of these elements suggest that both interfaces exhibited similar performance for imaging applications. Although the digital interface demonstrated superiority over the analog interface in terms of target tracking error, both interfaces exhibited comparable performance in resolution. This observation aligns with the experimental validation presented in the previous section.

Although the digital interface exhibited comparable optical resolution to the analog interface when images were reconstructed from the commanded scan waypoints, its more accurate position feedback becomes particularly beneficial for scan patterns that are physically challenging for the galvanometer to track. For example, the CLV-SC offers advantages over raster scanning by eliminating dead time while still providing uniform transverse sampling.^42^^,^^43^ However, it has also been reported to induce significant galvanometer position errors, particularly near the origin, due to a singularity problem.^20^^,^^44^^,^^45^ For such scan patterns, incorporating galvanometer position feedback is essential to reduce distortion and improve image quality near the spiral center, where the inherently high angular velocity of CLV-SC leads to tracking limitations.

**Fig. 11:**
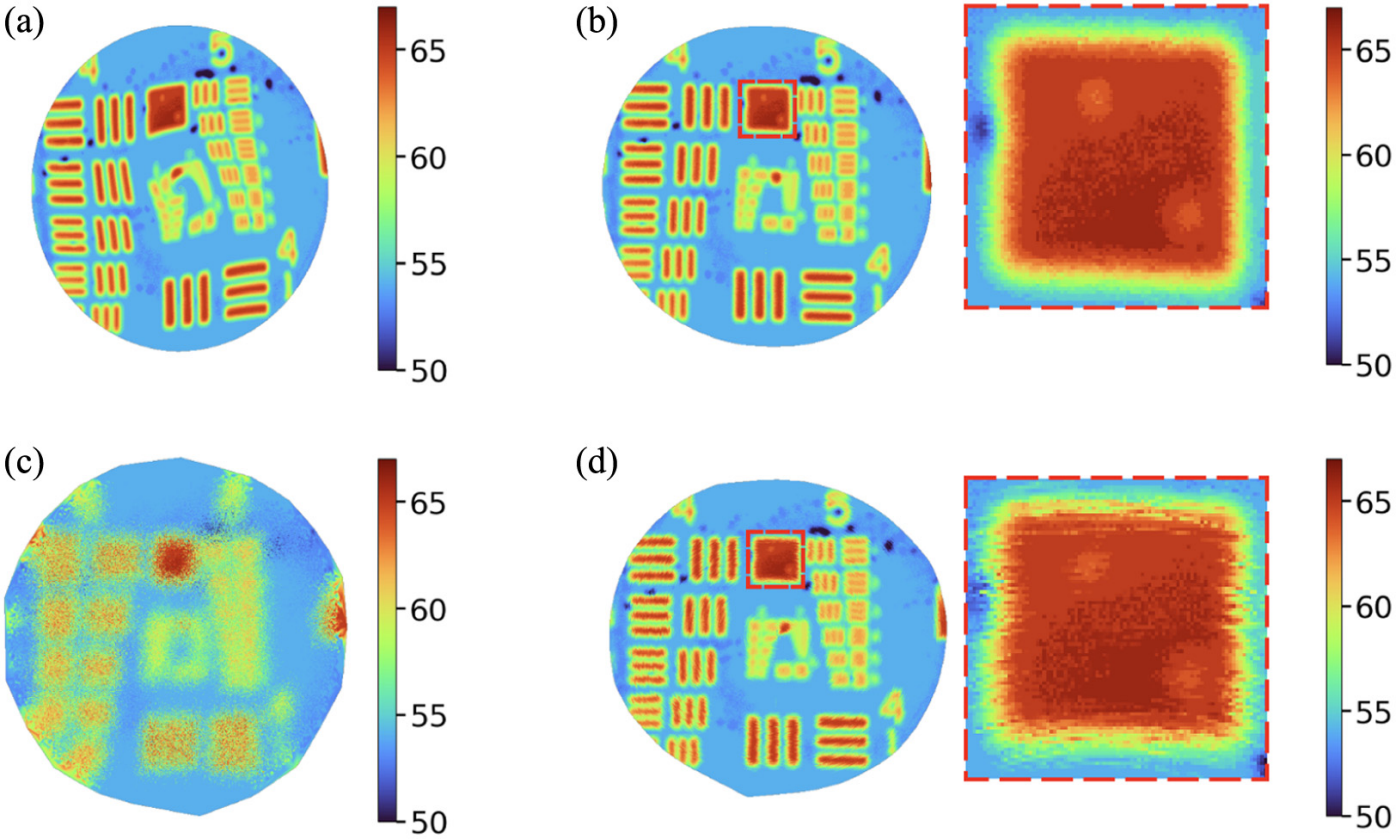
Comparison of CLV-SC reconstructions. (a) Reconstruction using the applied scan pattern, assuming perfect galvanometer tracking. (b) Reconstruction using position feedback from the digital interface. The inset highlights the square region between groups 4 and 5, showing a clear and well-defined boundary. (c) Reconstruction using position feedback from the analog interface (raw). (d) Reconstruction using analog feedback with Savitzky–Golay filtering applied to the position data (smoothed). The inset highlights the same region, exhibiting a comparatively blurred and noisy boundary relative to the digital feedback result.

Figure 11 shows reconstructed images of the USAF 1951 target generated from the galvanometer waypoints. Figure 11(a) illustrates the reconstruction based on the applied (commanded) waypoints, which is the conventional approach under the assumption that the actual galvanometer positions closely follow the commanded scan pattern. In the case of spiral scanning, however, this assumption does not hold, resulting in a significantly distorted target. Figure 11(b) presents the reconstruction based on the positions measured by the digital interface. Here, the distortion is largely corrected, and the resolution reaches group 5 element 3 for the horizontal features and group 5 element 4 for the vertical features, consistent with the resolution observed in Fig. 11(a). Figure 11(c) shows a highly blurred reconstruction based on analog position feedback in which the elements cannot be meaningfully resolved. This outcome reflects the high-noise nature of the analog position feedback and indicates that the analog interface is unsuitable as a complementary reconstruction standard for patterns such as spiral trajectories. Figure 11(d) displays the reconstruction after applying Savitzky–Golay filtering (window size of 300, polynomial order of 3) to the analog feedback signal. Although larger window sizes introduce noticeable image distortion (especially near the center and periphery), the chosen parameters improve the image quality compared with the raw analog case. Nonetheless, the resolution remains lower, and jitter-like artifacts persist, especially when compared with the reconstruction based on digital interface feedback.

### 5.5 Qualitative Imaging Results

**Fig. 12:**
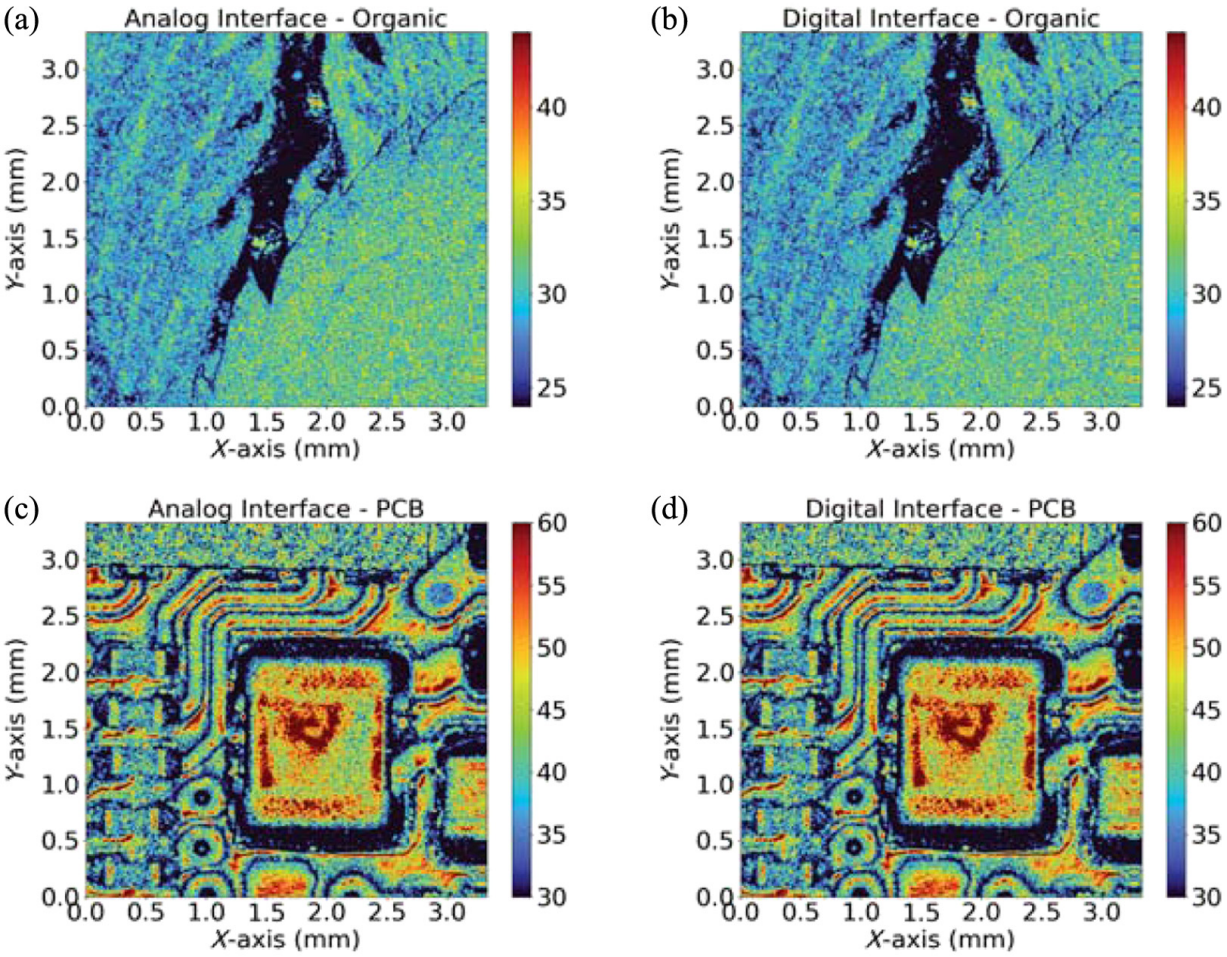
Representative images acquired using both analog and digital interfaces. (a) and (b) Leaves. (c) and (d) PCB components.

Qualitative equivalence was confirmed between the analog and digital interfaces. First, leaves from various plant species were scanned. As shown in Figs. 12(a) and 12(b), fine structural details such as venation and leaf tips were clearly visualized using both interfaces. Second, a PCB sample was imaged, given the widespread application of OCT in high-resolution inspection and metrology. Figures 12(c) and 12(d) demonstrate comparable visualization of electrical components, including resistors, capacitors, and conductive traces, obtained via both analog and digital interfaces.

**Fig. 13:**
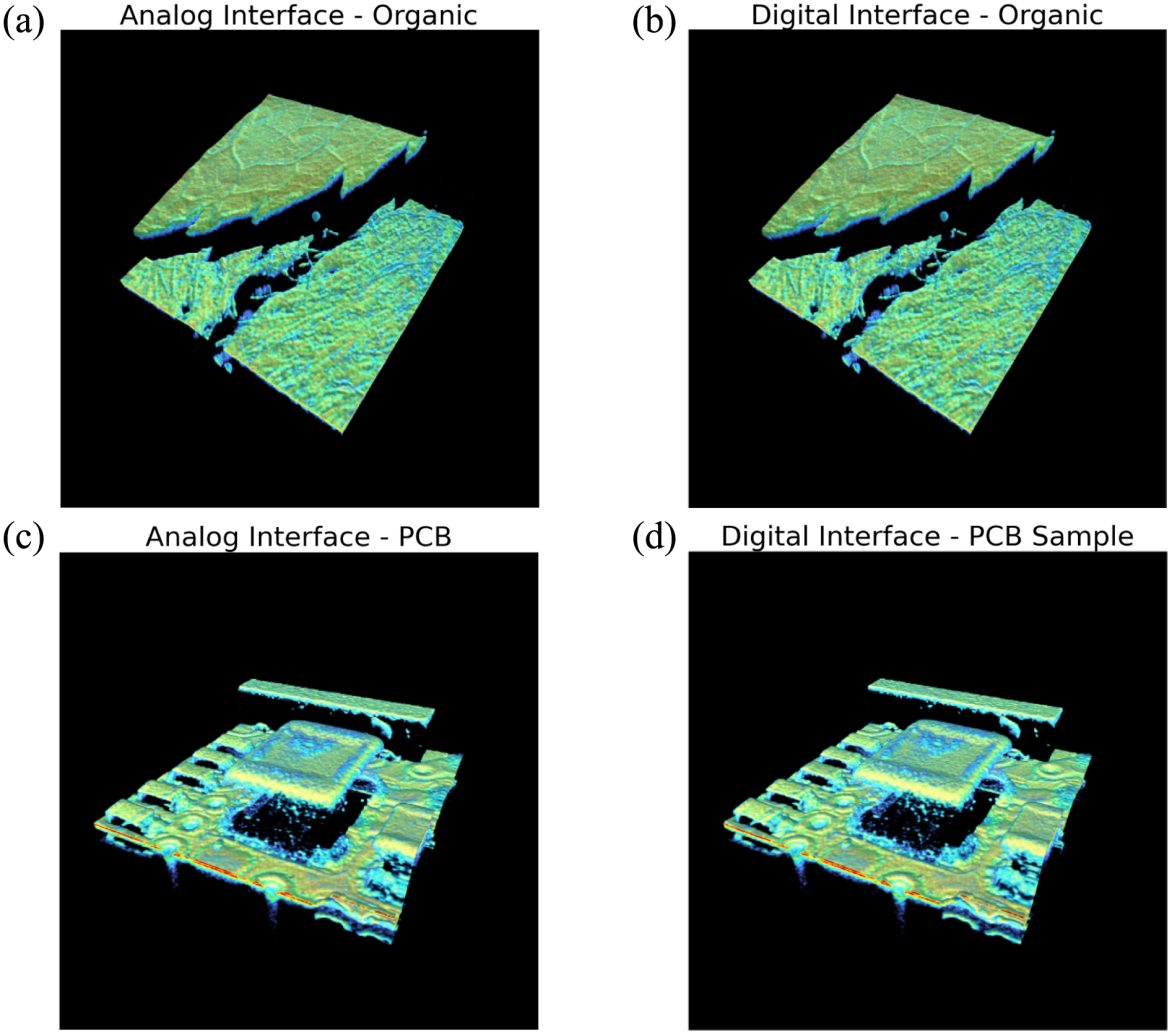
Representative 3D images acquired using both analog and digital interfaces. (a) and (b) Leaves. (c) and (d) PCB components.

Figure 13 illustrates the 3D reconstructions of OCT volumes acquired for each sample using both analog and digital interfaces. In the case of the organic sample [Figs. 13(a) and 13(b)], both reconstructions reveal multiple vertically stacked leaf layers, with venation patterns and leaf tips clearly preserved. For the PCB sample [Figs. 13(c) and 13(d)], both interfaces successfully capture the relative height differences among the electrical components.

## 6 Discussion

We present a compact, low-cost digital galvanometer control interface that delivers performance on par with, or superior to, traditional analog systems. Beyond demonstrating high positional accuracy and imaging consistency, the system exhibits enhanced robustness against analog-domain noise sources such as electromagnetic interference. Crucially, the digital design not only improves signal integrity but also reduces hardware complexity and cost by an order of magnitude while maintaining a smaller physical footprint. Together, these attributes make our digital interface a practical and scalable solution for precision imaging systems, offering reliable and efficient control without compromising performance. Furthermore, conventional digital protocols commonly used for galvanometer control, such as XY2-100 and SL2-100, are typically limited to 20-bit resolution (10 bits for *X* and *Y* positions each), which can constrain spatial accuracy in high-resolution imaging contexts. In contrast, our custom-built digital interface achieves an effective resolution of 32 bits (16 bits for *X* and *Y* positions each), offering up to 64-fold improvement in positional granularity. This enhanced resolution enables more accurate beam steering and contributes to the overall image quality required for advanced OCT applications. We have released software, schematics, and a bill of materials so that others can replicate this digital interface, as well as incorporate support for it in our open-source OCT library *vortex*.

Our investigation into signal noise revealed that the digital interface consistently outperformed its analog counterpart in terms of noise suppression. Nonetheless, position feedback analysis suggests that although the analog interface exhibited higher noise levels, this noise is likely filtered out by the servo driver, as evidenced by the similar noise profiles observed in both AWAR and DWAR signals. This implies that the position feedback error seen in the analog interface is introduced after the servo driver processes the feedback signal and does not accurately reflect the true position of the galvanometer. The noise observed in analog feedback is expected not to be directly linked to galvanometer movement but rather is introduced downstream, likely through susceptibility to electromagnetic interference (e.g., from a switching power supply) or signal degradation. Consequently, the actual galvanometer position, as regulated by the servo driver, is expected to be closely aligned with that of the digital interface. Also, laser-based raster scan imaging demonstrated that both interfaces produced comparable image quality, with no visually discernible differences. This observation suggests that the increased noise seen in analog position feedback is likely not representative of the galvanometer’s actual motion. We attribute this to the fact that the errors measured for both interfaces lie predominantly within the indistinguishable range of the current setup (−0.032 to 0.032 deg), such that differences between the two interfaces were not apparent in the acquired images.

We can also hypothesize that this discrepancy arises because the analog feedback signal, although noisier, is internally filtered by the servo driver before it is used to actuate the galvanometer. As a result, the true position of the galvanometer remains consistent with that driven by the digital interface, which benefits from intrinsically lower noise levels at the output stage. Another possible explanation is that the observed noise exceeds the bandwidth of the galvanometer, which inherently filters high-frequency fluctuations. In this case, even if the analog signal captures more noise, the galvanometer’s physical response may not follow those rapid variations, leading to a discrepancy between the “measured” and actual mechanical behavior. These results suggest that the galvanometer—specifically, the ScannerMax Saturn 1B model used in this study—exhibits high positional accuracy under typical raster scanning conditions. In fact, utilizing position feedback from an analog interface can degrade image quality compared with directly using the commanded scan pattern applied to the galvanometers. Unless the analog signal is heavily filtered and electronic noise is rigorously minimized, it is difficult to achieve image quality comparable to that obtained through reconstruction using the commanded scan pattern or the proposed digital interface. Therefore, careful selection of the signal source for image reconstruction is crucial—particularly in modalities sensitive to high-frequency noise of galvanometer positions, such as phase-sensitive OCT—to ensure high-fidelity scanning and precise phase measurement. Although this post-servo noise may not impact the physical accuracy of galvanometer motion, it can interfere with image reconstruction algorithms that depend on precise position feedback. Image reconstruction based on digital interface feedback outperformed reconstructions using either the analog interface or the ideal (commanded) scan pattern, underscoring its effectiveness for accurately capturing challenging and useful scan trajectories such as spiral scans. In this context, the digital interface proves advantageous by offering cleaner, more interpretable signals for position-dependent applications. In addition to its advantages in image quality for dynamically demanding scan patterns, the digital interface’s affordability and compact size make it a powerful complement to its analog counterpart in the field of OCT and biophotonics.

## 7 Conclusion

We developed a compact, low-cost, and robust digital interface for galvanometer control that achieves significantly improved accuracy compared with conventional analog systems, reducing positional feedback error by approximately half. Imaging experiments demonstrated comparable image quality between the two interfaces, whereas direct position tracking revealed that the analog feedback signal does not accurately represent the true galvanometer motion during scanning. Further experiments confirmed that the precise and stable feedback provided by the digital interface enables higher-quality image reconstruction, particularly for demanding scan patterns such as spiral scans. We believe this work establishes a foundation for the transition from analog to digital galvanometer control, offering a practical path toward more accurate and reliable position tracking of the galvanometer in OCT and broader biophotonics applications.

## 8 Appendix A

Table 2 summarizes the SAI configuration parameters in STM32 board that ensure compatibility with the FB4 communication protocol. 

**Table 2:** STM32 SAI settings for FB4 protocol compatibility.

Parameter	Value	Explanation
Clock Edge (CLK strobing)	Falling edge (low)	Data are captured or launched on the falling edge of the clock, aligning with the FB4 protocol
FrameSync (FS) pulse length	1 bit	Matches FB4 protocol, which uses one clock cycle for the FrameSync pulse
FrameSync (FS) polarity	Low	The FS line is driven low when FrameSync is active, just like in FB4
FrameSync (FS) offset	0	No offset between the FS pulse and data, aligning with FB4 timing
Slots	All active	All slots in the frame are considered active
Data size	32 bits	Each data word is 32 bits, compatible with the FB4 word size
Slot size	32 bits	Each slot is configured to 32 bits in length
Frame length	32 bits	One slot per frame, each 32 bits, matching the FB4 frame
SAI DMA functions	HAL_SAI_Transmit_DMA, HAL_SAI_Receive_DMA	Used in circular DMA mode to continuously transmit or receive data once started
SAI transmit callbacks	HAL_SAI_TxHalfCpltCallback, HAL_SAI_TxCpltCallback	Triggered by HAL_SAI_Transmit_DMA: HAL_SAI_TxHalfCpltCallback when half of the buffer is sent, HAL_SAI_TxCpltCallback when the entire buffer is sent
SAI/FB4 endianness	LSB first	For the SAI or FB4 protocol, data are transmitted with the least significant byte first. Computer-side byte order must be reversed, considering SPI endianness

## 9 Appendix B

Table 3 lists the UART and SPI communication settings between the STM32 microcontroller and the host computer.

**Table 3:** UART/SPI configuration and computer/STM32 settings.

Parameter	Value	Explanation
Computer software libraries	D2xx Driver, Windows.h, ftd2xx.h, libmpsse_spi.h	These drivers/headers must be installed or included for the operations on the computer side
SPI mode	Mode 2 (CPOL = 1, CPHA = 0)	SPI clock polarity is high (1) at idle, and data are captured on the first (leading) clock edge, based on ST Reference Manual RM0351
STM32 SPI configuration	Slave mode, mode 2	STM32 operates in SPI slave mode to match the computer master. Negative slave select (NSS) acts as the chip select (CS) line
STM32 SPI functions	HAL_SPI_Transmit_DMA (TX), HAL_SPI_Receive_DMA (RX)	When in slave mode, the STM32 uses DMA-based SPI transmission and reception
Computer SPI functions (C/C++)	SPI_Write (TX)	The computer sends data using SPI_Write and receives data using SPI_Read
	SPI_Read (RX)	
SPI endianness	MSB first	SPI transmits the most significant byte first
UART mode	8N1 (8 data bits, no parity, 1 stop bit)	This is the default format for many UART systems
STM32 UART functions	HAL_UART_Transmit_DMA(TX),	STM32 supports blocking and DMA-based UART communication, depending on throughput and latency requirements
	HAL_UART_Receive_DMA(RX),	
	HAL_UART_Transmit(TX),	
	HAL_UART_Receive(RX)	
Computer UART functions (C/C++)	WriteFile(TX),	UART communication on the computer side uses standard Windows serial APIs for asynchronous transmission and reception
	ReadFile(RX)	
Computer UART functions (Python)	serial.write(),	Python’s pyserial library is used for UART communication. Data are written via write() and read via read() or readline()
	serial.read(),	
	serial.readline()	

## 10 Appendix C

The TIM interface continuously generates pulses at specified intervals, with its frequency determined by a user-defined counter period. When the counter reaches that period, it resets and emits a new pulse. A key feature is its ability to reset upon receiving an external trigger, allowing the timer to restart immediately regardless of its current count. Using the laser trigger as the external source, the TIM interface can produce both the FrameSync and clock signals needed for SAI transmission and reception through a combination of downsampling and upsampling of the laser trigger. Initially, a PWM signal is generated for FrameSync. By employing the DMA channel of the TIM interface, we produce a continuous PWM signal with varying pulse widths that can act as FrameSync of the SAI channel. Subsequently, we generate the clock signal, triggered internally by the TIM channel that is used for FrameSync generation. Equation (3) illustrates how the TIM interface operates in the time domain. In this equation, CP stands for the counter period, PC for the peripheral clock period, and PS represents the prescaler utilized in the interface. ACP stands for actual counter period in seconds. The peripheral clock of the TIM interface is connected to the internal clock bus, APB2, of the STM32 board, with a frequency of 80 MHz, which makes *PC* 0.0125  μs. The values for PS and CP are incremented by 1 because these parameters are set in the STM32 interface as zero-based

ACP=PC*(PS+1)*(CP+1). (3)

For the FrameSync channel of the timer, the prescaler is set to 9 (10 − 1). The pulse configuration is set to 1, 0, 0, 0, resulting in a pulse length of 0.125  μs. This configuration implies that it will take four clock periods to generate the next pulse, as only one pulse is non-zero among the four pulses. However, to synchronize with the external trigger signal, the timer must reset before it reaches the internal counter period. To satisfy this condition, the counter period must be set longer than the period of the laser trigger. In our system, the clock period is set to 255 (256 − 1) for the FrameSync channel of the timer, allowing it to operate with an external trigger down to 31.25 kHz.

For the clock channel of the timer, the prescaler is set to 4 (5 − 1). The pulse length is configured to 1, and the clock period is set to 1 (2 − 1). According to Eq. (3), the clock signal will have a period of 0.125  μs and a duty cycle of 50 %, which is typical for a clock signal. The clock period is set to be the same as the pulse length of FrameSync, considering the ideal pulse of FrameSync for the FB4 protocol is the period of the clock. The clock channel of the timer is internally triggered by the reset of the FrameSync channel. The polarity for both signals is set to high to be compatible with the FB4 protocol.

## 11 Appendix D

**Fig. 14:**
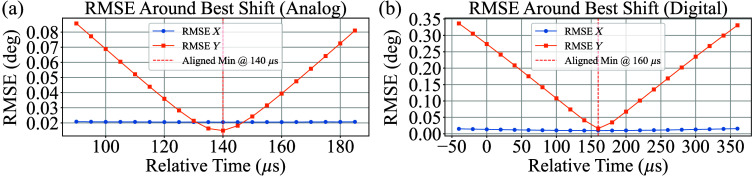
(a) RMSE values for X and Y positions computed across time shifts of the analog signal, with minimum alignment error observed at 140  μs. (b) RMSE values computed for digital signal alignment, showing a minimum at 160  μs.

Figure 14 shows RMSE of X and Y alignments across relative time shifts for both analog and digital systems. It shows that the optimal alignment occurs at 140 µs for the analog configuration and 160 µs for the digital configuration, where the RMSE reaches its minimum.

## Data Availability

The source code, PCB schematic, bill of materials, and user manual for the digital scanner interface are available at https://github.com/igmr-lab/Digital_Galvo The datasets supporting the findings of this study are currently not publicly accessible but can be provided by the authors upon reasonable request.
